# Neurofibromin 1 alters feeding microstructure in *Drosophila*

**DOI:** 10.17912/micropub.biology.002374

**Published:** 2026-10-21

**Authors:** Zohre Panahidizjikan, Sydney Born, Maia Espinoza, Tate Lillian, Elizabeth B Brown

**Affiliations:** 1 Biological Sciences, Florida State University, Tallahassee, FL, United States; 2 Miami Palmetto Senior High School, Pinecrest, FL, United States; 3 Young Scholars Program, Florida State University, Tallahassee, FL, United States; 4 Magnet Innovation Center, Inlet Beach, FL, USA; 5 Program in Neuroscience, Florida State University, Tallahassee, FL, United States

## Abstract

Neurofibromatosis type 1 (NF1) is caused by the loss of neurofibromin, a protein shown to regulate metabolism and feeding behavior in *Drosophila melanogaster.* Using the FlyPad two-choice feeding assay, we compared feeding microstructure between *NF1* null mutants, NF1 heterozygotes, and wildtype controls. We find that *NF1* mutants took significantly more sips of sucrose and with greater urgency, without changes in sip duration or sucrose taste sensitivity. Overall, these results suggest loss of *NF1* increases feeding frequency.

**Figure 1. Loss of NF1 increases food intake:** **(A)** Schematic of the FlyPad two-choice feeding assay, in which individual flies are offered simultaneous access to 1mM and 5mM sucrose. **(B)** There is a significant effect of genotype on sip number (H test: H _2_ = 23.54, *P* <0.0001). Loss of *NF1* significantly increases sip number (+, *P* <0.0001; het, *P* <0.0085). **(C)** There is a significant effect of genotype on sip number when binned by sucrose concentration (REML: F _2,268_ = 11.00, *P* <0.0001). Loss of *NF1* has no effect on sips of 1mM sucrose (+, *P* <0.3706; het, *P* <0.3915), but significantly increases sips of 5mM sucrose (+, *P* <0.0001; het, *P* <0.0149). **(D)** Loss of *NF1* has no effect on preference for 5mM sucrose (H test: H _2_ = 4.470, *P* <0.1070). **(E)** There is no effect of genotype on sip duration (REML: F _2,134_ = 2.895, *P* <0.0588). **(F)** There is no effect of genotype on intersip interval (REML: F _2,132_ = 1.253, *P* <0.2890). **(G)** There is a significant effect of genotype on feeding intensity (REML: F _2,260_ = 13.25, *P* <0.0001). Loss of *NF1* has no effect on sips of 1mM sucrose (+, *P* <0.3399; het, *P* <0.5110), but significantly increases sips of 5mM sucrose (+, *P* <0.0001; het, *P* <0.0001). **(H-J)** Distribution of the durations of sips of sucrose in **(H)** wildtype flies, **(I)** *NF1* heterozygous flies, and **(J)** *NF1* mutant flies. Error bars indicate ± SEM. * *p* <0.05; ** *p* <0.01; **** *p* <0.0001.

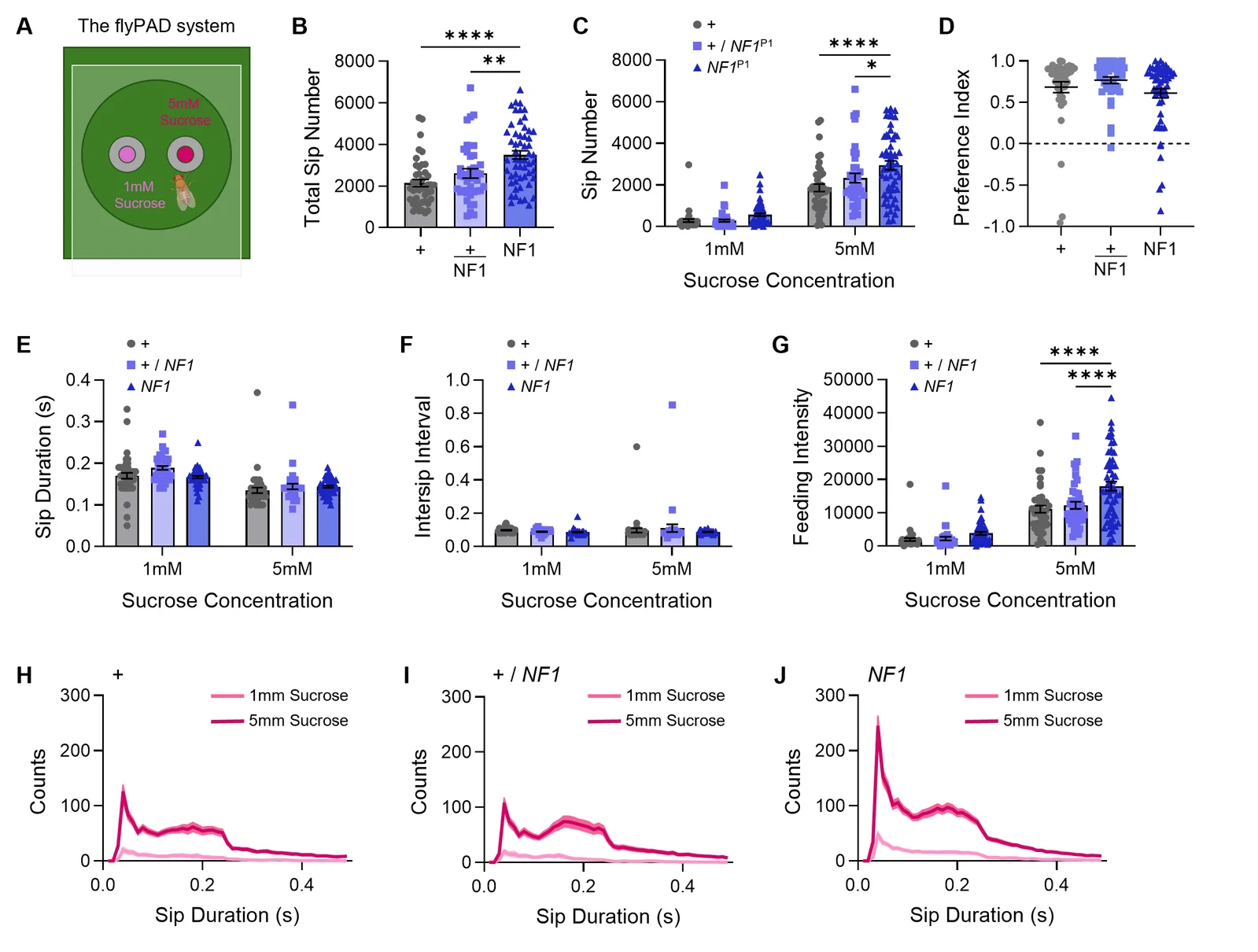



## Description

Neurofibromatosis type 1 (NF1) is a neurodevelopmental disorder caused by mutations in the *NF1* gene. This gene encodes neurofibromin, a GTPase-activating protein that negatively regulates the RAS signaling pathway (Buchberg et al., 1990; Xu et al., 1990). In humans, mutations in *NF1* are associated with disruptions in several physiological and behavioral traits, including body size, metabolic function, and behavior (Botero & Tomchik, 2024). Many aspects of this disorder can be phenocopied using the fruit fly, *Drosophila melanogaster* : *NF1* -deficient flies have reduced body size, elevated metabolic rate, and reduced triglyceride stores (Botero et al., 2021, 2026; Brown et al., 2023; Sofela et al., 2024; The et al., 1997; Walker et al., 2006). Notably, loss of *NF1* increases food intake (Botero et al., 2021; Sofela et al., 2024), consistent with a homeostatic response to their elevated metabolic rate and depleted energy stores. In this study, we further characterized the feeding behavior of *NF1* flies.

Several approaches have been developed to quantify feeding behavior in *Drosophila* . These include the capillary feeder (CAFE) assay, which measures cumulative food consumption from a graduated capillary (Ja et al., 2007), manual scoring of proboscis extensions (Mair et al., 2005; Wong et al., 2009), as well as dye-labeling and radioisotope-labeling based approaches that estimate ingestion from colorimetric food additives or radiolabeled food, respectively (Carvalho et al., 2005; King & Wilson, 1955; Shimada et al., 1987; Tanimura et al., 1982). These approaches provide robust measures of total intake but lack the temporal and behavioral resolution to resolve individual feeding events. We therefore used the flyPAD ( fly P roboscis and A ctivity D etector), which can detect each contact between a fly’s proboscis and a food source as a discrete change in capacitance, thereby enabling real-time, high-resolution quantification of feeding microstructure including sip number, duration, and choice between food sources in individual, freely feeding flies (Itskov et al., 2014).

Here, we utilized the flyPAD to investigate the effects of *NF1* on food intake and feeding microstructure ( **Fig 1A** ). Starved male flies were provided the opportunity to feed from 1 mM and 5 mM sucrose, a paradigm that reports both feeding motivation and the ability to discriminate caloric value (Collins et al., 2026). We used the *NF1* ^P1^ mutant line, which has a deletion that removes all of the *NF1* gene except for the first exon (The et al., 1997). We found that when we summed the total number of sips taken from both wells over the duration of the experiment *NF1* ^P1^ mutant flies take significantly more sips than both control and *NF1* ^P1^ heterozygote flies ( **Fig 1B** ), consistent with previous findings that disruption of NF1 increases food intake (Botero et al., 2021; Sofela et al., 2024). We next broke down the number of sips by sucrose concentration and found that all genotypes took more sips of 5mM sucrose than of 1mM sucrose. However, *NF1* ^P1^ mutant flies consistently took more sips of 5mM sucrose relative to control and *NF1* ^P1^ heterozygote flies ( **Fig 1C** ). To evaluate the possibility that *NF1* ^P1^ mutant flies are more sensitive to 5mM sucrose, we calculated a Preference Index for 5mM sucrose, where a positive value indicates stronger preference for 5mM sucrose and a negative value indicate stronger preference for 1mM sucrose. We found that preference for 5mM sucrose was consistent across all genotypes ( **Fig 1D** ), suggesting taste sensitivity to sucrose does not mediate the increased sips taken by *NF1* ^P1^ flies.

In wild-type flies, food deprivation alters the microstructure of feeding by increasing the total number and duration of sips, and by decreasing the length between sips (Itskov et al., 2014; Banu et al., 2023). We next investigated whether NF1 functions to regulate these additional metrics of feeding microstructure. We found that the duration of each sip was not significantly different between genotypes ( **Fig 1E** ), nor was the interval between sips ( **Fig 1F** ). Therefore, *NF1* ^P1^ flies consume more 5mM sucrose by increasing the frequency of sipping, not by taking longer sips or reducing time between sips, suggesting that loss of *NF1* engages a more restricted feeding-drive mechanism than food deprivation alone. This is further reflected in measurements of feeding urgency (Collins et al., 2026), where we find that *NF1* ^P1^ flies have significantly higher feeding intensity on 5mM sucrose relative to control and *NF1* ^P1^ heterozygotes ( **Fig 1G** ). This may be driven primarily by the observation that *NF1* ^P1^ flies have a greater frequency of very short duration sips relative to control and heterozygotes ( **Fig 1H-J** ). Overall, our results suggest that loss of *NF1* further increases feeding frequency without altering the duration of individual sipping episodes or the ability to discriminate between sucrose concentrations, consistent with an increase in feeding drive.

## Methods

*Drosophila care and maintenance*

Flies were maintained on standard cornmeal-molasses-yeast food (Bloomington Recipe, Genesee Scientific, San Diego, CA) at 25°C and at ~50% relative humidity under a 12 h light: 12 h dark cycle. Mated ~3–5-day-old male flies were collected under light CO _2_ anesthesia and allowed to recover for at least 24 h prior to all experiments.

*FlyPAD two-choice feeding assay*

Feeding behavior was quantified using the flyPAD (fly Proboscis and Activity Detector; Itskov et al., 2014). Prior to testing, flies were starved for 18 h in vials on wet Kimwipe paper. On the day of testing, individual males were aspirated without anesthesia into flyPAD arenas configured for a two-choice assay, with the two food channels containing either 1 mM or 5 mM sucrose in 1% agarose. Flies were allowed to feed for 60 min, after which data were extracted and analyzed using established flyPAD analysis software (Itskov et al., 2014). The Preference Index (PI) was calculated as: (# sips of 5 mM sucrose − # sips of 1 mM sucrose) / (total # of sips), where a value of 1.0 indicates complete preference for 5mM sucrose and a value of -1.0 indicates a complete preference for 1mM sucrose. Feeding Intensity was calculated as: # sips / sip duration (Collins et al., 2026). Flies that did not feed were excluded from downstream analyses. The resulting sample size for this experiment were as follows: controls (N = 43), *NF1* ^P1^ heterozygotes (N = 39), and *NF1* ^P1^ mutants (N = 55). All experiments were performed between ZT 0-2 at 25°C.

*Statistical analysis*

Feeding metrics are visualized as graphs displaying mean ± standard error. Dots represent individual fly measurements. Four independent runs were performed. The data were not normally distributed and so the non-parametric Kruskal-Wallis test was used to compare genotypes. To compare genotypes and sucrose concentration, a restricted maximum likelihood (REML) estimation was used. All *post hoc* analyses were performed using Dunn’s multiple comparisons test. Data were analyzed and visualized using InStat software (GraphPad Software 11.0).

## Reagents

*Drosophila lines*

The control line, *w* ^1118^ was obtained from the Bloomington Stock Center (#5905). The *NF1* ^P1^ line was a gift from Seth Tomchik’s lab at the University of Iowa (The et al., 1997). This line was backcrossed to the *w* ^1118^ laboratory strain for 10 generations prior to testing.
